# Ceramic Perovskite-Based Photoelectrochemical Platform for 2‑(3,4-Dihydroxyphenyl)ethylamine Detection with Enhanced Sensitivity

**DOI:** 10.1021/acsomega.5c02185

**Published:** 2025-07-04

**Authors:** Lenilda Ferreira Costa, José Ribamar Nascimento dos Santos, Jefferson Santos Oliveira, Alan Silva de Menezes, Gilvan Pereira de Figueredo, Flávio Santos Damos, Rita de Cássia Silva Luz

**Affiliations:** † Laboratory of Sensors, Devices and Analytical Methods, Federal University of Maranhão, 65080-805 São Luís, MA, Brazil; ‡ Postgraduate Program in Chemistry Associative UFMA-IFMA, Federal Institute of Education, Science and Technology of Maranhão, 65030-005 São Luís, MA, Brazil; § Postgraduate Program in Physics, CCET, Federal University of Maranhão, 65080-805 São Luís, MA, Brazil

## Abstract

2-(3,4-Dihydroxyphenyl)­ethylamine, DPE, is a critical catecholamine that regulates vital physiological functions, and its dysregulation is associated with heart disease, hypertension, kidney failure, and neurological disorders, highlighting the need for reliable detection methods. Ceramic perovskites, characterized by their unique crystal structure, exhibit remarkable properties that make them highly suitable for technological applications. In this study, we explore the synergistic combination of two zirconium-based ceramic perovskites—barium zirconate (BaZrO_3_) and strontium zirconate (SrZrO_3_)—for the development of a photoelectrochemical platform designed to detect DPE. A fluorine-doped tin oxide (FTO) electrode was modified with materials based on BaZrO_3_ and SrZrO_3_ to create a highly efficient photoelectrochemical sensor. The combination of these materials significantly enhanced the sensor’s performance compared to their individual use, facilitating faster electron transfer rates and improved sensitivity for DPE detection. A low-power LED lamp served as the light source, ensuring energy efficiency. The materials were synthesized by microwave-assisted combustion and thoroughly characterized using X-ray diffraction (XRD), Fourier-transform infrared spectroscopy (FTIR), scanning electron microscopy (SEM), energy-dispersive X-ray spectroscopy (EDS), and chronoamperometry. Under optimized conditions, the sensor demonstrated two linear detection ranges for DPE: 0.01 to 100 μmol L^–1^ and 100 to 500 μmol L^–1^, with a detection limit of 0.009 μmol L^–1^. The modified platform (SrZrO_3_/BaZrO_3_/FTO) exhibited exceptional precision, accuracy, and selectivity for DPE detection. Furthermore, the method was successfully applied to determine DPE concentrations in artificial plasma samples, achieving recovery rates between 98.32 and 102.13%. These results highlight the promising character of this photoelectrochemical platform for reliable and sensitive DPE detection in real-world applications.

## Graphical abstract

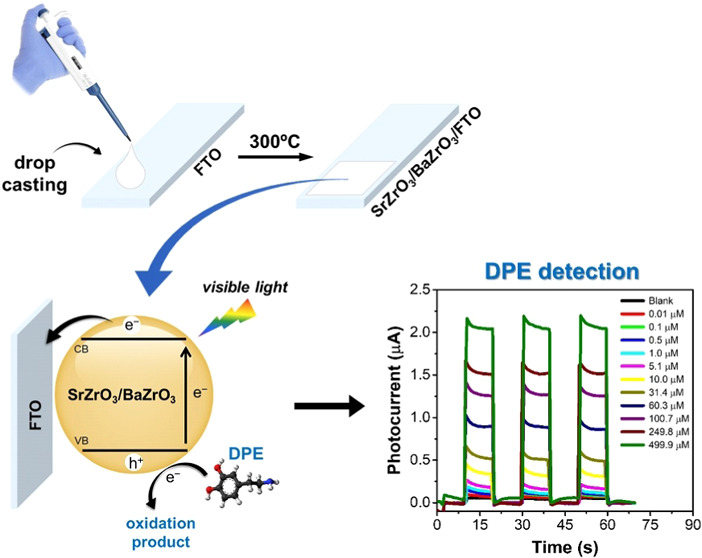

## 1 Introduction

The search for precise and sensitive analytical methods for the detection of essential biomolecules, such as catecholamines, has gained prominence in the scientific landscape due to their crucial role in the diagnosis and monitoring of various diseases.^1^ Among these biomolecules, 2-(3,4-dihydroxyphenyl)­ethylamine (DPE), commonly known as dopamine, emerges as a highly relevant target.^1^ DPE not only regulates fundamental physiological functions, such as motor control, mood, and cognition, but also acts as a key neurotransmitter in the central nervous system.^2^ Changes in its levels are directly associated with a variety of pathologies, including cardiovascular diseases, hypertension, kidney failure, and neurological disorders such as Parkinson’s and schizophrenia.^3^ Given its importance, the reliable and sensitive detection of DPE becomes an urgent necessity for advancing therapeutic and diagnostic strategies.

Several methods have been developed for the detection of DPE, each with its own advantages and limitations. The most commonly used techniques include high-performance liquid chromatography (HPLC) coupled with ultraviolet (UV) or with electrochemical detection, which is considered the gold standard for DPE quantification due to its high selectivity and accuracy. However, HPLC requires expensive equipment, skilled operators, and time-consuming sample preparation, limiting its use in point-of-care applications.^4,5^

Fluorescence and UV–vis spectroscopy rely on the interaction of DPE with specific probes or reagents that produce a measurable signal. While sensitive, they often require complex derivatization steps and are prone to interference from matrix effects.^6,7^ Enzyme-linked immunosorbent assays (ELISAs) and other immunoassay-based methods offer high specificity for DPE detection. However, they are expensive, time-consuming, and require specialized antibodies, making them less suitable for rapid and large-scale screening.^8,9^ Despite their widespread use, these conventional methods often face challenges such as low selectivity, high cost, and the need for complex instrumentation. These limitations have driven the search for alternative approaches that combine high sensitivity, selectivity, low-cost, and practicality.^10^

Electrochemical Methods are also widely employed due to their high sensitivity, simplicity, and low cost.^10^ Techniques such as cyclic voltammetry (CV) and amperometry are frequently used to detect DPE based on its redox activity. However, these methods often suffer from interference from other electroactive species, such as ascorbic acid and uric acid, which coexist with DPE in biological fluids.^11^ While traditional literature often focuses on conventional electrochemical methods for DPE detection, this work proposes an innovative approach by integrating ceramic perovskites into a photoelectrochemical platform.

Barium Zirconate (BaZrO_3_) is known for its high chemical stability and wide band gap (∼5.0 eV), making it an excellent candidate for applications requiring robust materials under harsh conditions. Its cubic perovskite structure provides a stable framework that can be functionalized to enhance its photoelectrochemical properties.^12^ Strontium Zirconate (SrZrO_3_) also exhibits a wide band gap (∼5.6 eV) and shares similar stability characteristics.^13^ However, its slightly different ionic radius and electronic configuration can lead to variations in charge carrier dynamics, which can be exploited to optimize photoelectrochemical performance. Furthermore, the BaZrO_3_ and SrZrO_3_ have exhibited exceptional recyclability, underscoring its potential as environmentally friendly photoelectrochemical materials.^12,13^ However, the use of these materials in photoelectrochemical systems has been limited due to their high band gaps, which restrict the absorption of visible light and, consequently, the generation of photocurrent. To address this, we have developed a novel strategy that involves the interaction of these perovskites with organic molecules that act as charge transfer species. This approach may not only broaden the light absorption range but also enhances the efficiency of photoelectrochemical performance, enabling the detection of DPE with sensitivity and selectivity superior to those achieved by traditional methods.

The integration of BaZrO_3_ and SrZrO_3_ into a single platform leverages the complementary properties of these materials. BaZrO_3_ provides a stable and robust material, while SrZrO_3_ offers enhanced electronic properties that can facilitate faster electron transfer rates. Thus, such combination can synergistically improve the performance of photoelectrochemical sensors, including higher sensitivity and lower detection limits for target analytes.

This work not only advances the state of the art in DPE detection but also opens new perspectives for the use of ceramic perovskites in development of photoelectrochemical platforms. Recent studies have highlighted the potential of perovskite-based materials for the development of highly efficient and practical sensors, paving the way for their application in clinical diagnostics and health monitoring.

## 2 Experimental Section

### 2.1 Reagents

All reagents used were of analytical grade and used without additional purification steps. All solutions were prepared with deionized water. Barium nitrate was obtained from Dinâmica Química (Indaiatuba, São Paulo, Brazil), pentahydrated zirconium nitrate from INLAB (São Paulo, Brazil), strontium nitrate (Synth), and urea from Neon (Suzano, São Paulo, Brazil). DPE, sucrose, glucose, fructose, ascorbic acid, magnesium chloride, 2-[4-(2-hydroxyethyl)­piperazine-1-yl]­ethanesulfonic acid (HEPES), acetic acid (CH_3_COH), sodium hydroxide (NaOH), sodium sulfite, citric acid, and potassium chloride were obtained from Sigma-Aldrich (São Paulo, Brazil). Monobasic sodium phosphate, sodium chloride, boric acid (H_3_BO_3_), phosphoric acid (H_3_PO_4_), potassium chloride, magnesium chloride, calcium chloride, sodium sulfate, anhydrous dibasic sodium phosphate, and sodium bicarbonate were obtained from Isofar (Duque de Caxias, Rio de Janeiro, Brazil).

### 2.2 Synthesis of BaZrO_3_ and SrZrO_3_

BaZrO_3_ and SrZrO_3_ based-powders were synthesized by microwave-assisted combustion, according to procedures adopted for the synthesis of perovskite-type oxides^14^ and based on the general principles of the chemistry of explosives and propellants.^15^ Initially, for the synthesis of BaZrO_3_, 0.4773 g of Ba­(NO_3_)_2_, 0.7840 g of Zr­(NO_3_)_2_·5H_2_O, and 0.5484 g of (NH_2_)_2_CO were weighed. For the synthesis of SrZrO_3_, 0.4712 g of Sr­(NO_3_)_2_, 0.9558 g of Zr­(NO_3_)_2_·5H_2_O, and 0.6686 g of (NH_2_)_2_CO were weighed. The nitrates and urea were dissolved in 12 mL of distilled water and heated to 60 °C until complete dissolution. The resulting solutions were heated in an adapted microwave oven with a frequency of 3.45 GHz and a power of 900 W for less than 5 min until the combustion reaction was complete. To uniformize the phase of the samples, the obtained solids were calcined at 900 °C for 3 h with a heating rate of 10 °C min^–1^ in a muffle furnace.

### 2.3 Characterizations and Electrochemical Measurements

To characterize the crystal structure of the materials used in constructing the photoelectrochemical (PEC) platform, X-ray diffraction (XRD) analyses were performed using a SHIMADZU XRD-6100 diffractometer equipped with a Cu Kα radiation source (λ = 1.5406 Å) operating at 30 kV and 30 mA. The measurements were conducted over an angular range of 10–80° (2θ) with a scanning speed of 2° min^–1^ and a step size of 0.02°. Phase quantification, lattice parameters, and other microstructural properties were determined through Rietveld refinement using the GSAS 2 (General Structure Analysis System) software. The crystallite size was calculated using the Scherrer eq (eq 1):^16^

1 $$D_{\mathrm{HKL}}=\frac{0.9\lambda }{\beta \;\cos\theta }$$

where *D*_HKL_ is the crystallite size in the HKL direction, λ is the wavelength of the X-ray radiation, β is the instrumental broadening factor, and θ is the Bragg angle corresponding to each diffraction peak.

Fourier-transform infrared (FTIR) spectroscopy was employed to analyze the chemical bonding and functional groups present in the synthesized materials. The spectra were acquired using a Shimadzu IR Prestige-21 spectrometer in the range of 400–4000 cm^–1^. Samples were prepared as KBr pellets (1% sample concentration) to ensure optimal signal detection.

Morphological and compositional analyses were performed using scanning electron microscopy (SEM) and energy-dispersive X-ray spectroscopy (EDS). SEM images were obtained using a Zeiss EVO HD detector, while EDS measurements were conducted with a Bruker XFLASH 410 detector to determine the elemental composition of the materials. Au layer was deposited, using a Denton Vacuum DESK IV Sputter Coater (40 mA for 1 min) to ensure conductivity and avoid charging effects. Gold film was deposited via sputtering on precleaned substrate treated with O_2_ plasma.

Electrochemical experiments were carried out at atmospheric pressure and room temperature using a three-electrode configuration controlled by a PGSTAT128N potentiostat/galvanostat (Metrohm-Autolab) and NOVA 2.1 software. To ensure controlled light exposure, the electrochemical cell was placed inside a homemade light-tight box. A 36 W LED lamp served as the visible light source for photoelectrochemical measurements. The working electrode consisted of an FTO glass substrate modified with SrZrO_3_/BaZrO_3_ (5 cm × 1 cm, with an active area of 0.7 × 0.9 cm^2^). An Ag/AgCl/KCl_saturated_ electrode was used as the reference electrode, and a gold wire acted as the auxiliary electrode. Electrochemical impedance spectroscopy (EIS) experiments were performed in 0.1 mol L^–1^ PBS (phosphate buffer solution) in the absence and in the presence of 1 mmol L^–1^ of DPE under LED illumination in a frequency range from 10 kHz to 0.1 Hz.

### 2.4 Construction of the SrZrO_3_/BaZrO_3_/FTO Platform

To modify the FTO substrate, 3.0 mg of the sample containing SrZrO_3_ and 2.0 mg of the sample containing BaZrO_3_ were mixed in 20 μL of deionized water and then homogenized using ultrasound for 10 min. From this suspension, 20 μL were taken and directly deposited onto the surface of the FTO electrode. The electrode was allowed to dry at room temperature for 30 min and then heated on a hot plate at 300 °C for 30 min, thus forming a film composed of BaZrO_3_/SrZrO_3_ material on the FTO surface (SrZrO_3_/BaZrO_3_/FTO platform).

### 2.5 Preparation of Artificial Plasma Samples

Artificial plasma samples were prepared according to the procedure described in the literature.^17^ The aqueous dispersion of artificial human plasma was prepared in deionized water, with the chemical composition of NaCl, KCl, MgCl_2_, CaCl_2_, Na_2_SO_4_, Na_2_HPO_4_ and NaHCO_3_ at the respective concentrations (8.036, 0.225, 0.145, 0.293, 0.072, 0.076, and 0.325 g L^–1^).^17^ After preparation, the artificial plasma was stored in a transparent glass vial and kept refrigerated at approximately 4 °C. Each sample was fortified with a different concentration of DPE for subsequent analysis in the electrochemical cell containing 5 mL of 0.1 mol L^–1^ PBS (pH 7.0).

### 2.6 Analytical Curve

The analytical curve was constructed by successive additions of aliquots of DPE stock solutions into an electrochemical cell initially containing 5.0 mL of 0.1 mol L^–1^ PBS (pH 7.0). The stock solutions, prepared by diluting the analyte in the same PBS buffer, covered a concentration range from 5 × 10^–6^ mol L^–1^ to 1 × 10^–2^ mol L^–1^. By adding varying aliquots of these solutions to the cell, final DPE concentrations spanning 0.01 to 500 μmol L^–1^ were achieved. The evaluation of the SrZrO_3_/BaZrO_3_/FTO sensor included the determination of DPE in both artificial plasma matrix and clinical osteoporosis tablets, using external calibration.

## 3 Results and Discussion

### 3.1 Structural Characterization

The XRD patterns obtained for BaZrO_3_, SrZrO_3_, and the SrZrO_3_/BaZrO_3_ based-samples are presented in Figure 1. The diffraction patterns for BaZrO_3_ (Figure 1a) and SrZrO_3_ (Figure 1b) are consistent with the data from the JCPDS standards 00-006-0399 (BaZrO_3_) and 01-075-0467 (SrZrO_3_), both exhibiting a cubic perovskite structure with the space group *Pm3̅m*.

**Figure 1:**
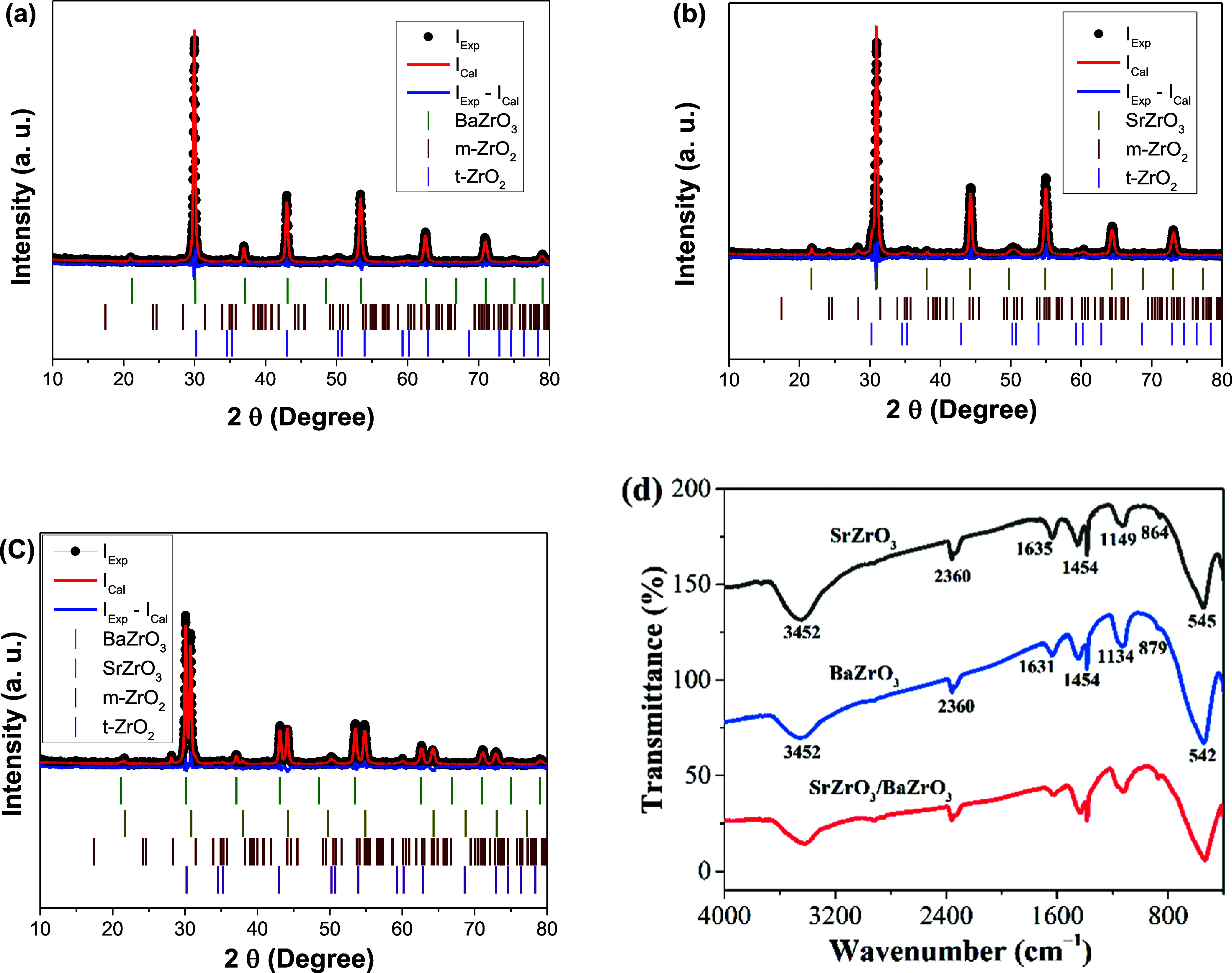
X-ray diffraction patterns for BaZrO_3_ (a), SrZrO_3_ (b), and SrZrO_3_/BaZrO_3_ (c). (d) FTIR spectra obtained for SrZrO_3_, BaZrO_3_, and SrZrO_3_/BaZrO_3_.

The XRD data analysis indicates that BaZrO_3_ based-sample is composed of a mixture of the BaZrO_3_ perovskite phase (JCPDS 00-006-0399) and two forms of zirconium oxide: monoclinic (m-ZrO_2_) (JCPDS 01-080-0966) and tetragonal (t-ZrO_2_) (JCPDS 01-080-0965). To identify the phases present and determine the crystallographic parameters, Rietveld refinement was performed, as shown in Figure 1a and Table 1. The refinement yielded values of Goodness of Fit (S) = 1.1 and *R*_wp_ = 15.3%, which are considered satisfactory for phase confirmation. The barium zirconate perovskite phase is predominant, with a phase fraction of 91.4%, while monoclinic and tetragonal zirconium oxide constitute 2.4 and 6.2% of the system, respectively. Table 1 also presents the obtained crystallite size values, which indicate that both phases have nanometric sizes.

**Table 1:** Crystallographic Parameters from Rietveld Refinement for BaZrO_3_ and SrZrO_3_

		**experimental**							
**phase**	**space group**	***a* (Å)**	***b* (Å)**	***c* (Å)**	**β (** ^**o**^ **)**	**phase fraction**	***D* (nm)**	***R***_**wp**_ **(%)**	***S***
**BaZrO**_**3**_	cubic *Pm*3̅m	4.193(2)				91.4(4)	54(1)	15.3	1.1
**m-ZrO**_**2**_	monoclinic *P*21/*c*	5.16(2)	5.24(2)	5.28(3)	99.0(1)	2.4(4)	64(2)		
**t-ZrO**_**2**_	tetragonal *P*42/*nmc*	3.600(2)		5.185(3)		6.2(4)	83(9)		
**SrZrO**_**3**_	cubic *Pm*3*®m*	4.096(1)				84.4(4)	54.8(1)	14.9	1.3
**m-ZrO**_**2**_	monoclinic *P*21/*c*	5.19(2)	5.227(2)	5.27(2)	98.47(9)	6.9(4)	51(9)		
**t-ZrO**_**2**_	tetragonal *P*42/*nmc*	3.592(1)		5.183(2)		8.7(3)	51(5)		
**BaZrO**_**3**_	cubic *Pm*3̅*m*	4.189(1)				38.2(3)	66(2)	14.0	1.2
**SrZrO**_**3**_	cubic *Pm*3̅*m*	4.098(1)				48.8(3)	60(1)		
**m-ZrO**_**2**_	monoclinic *P*21/*c*	5.149(7)	5.202(3)	5.320(7)	99.16(4)	4.9(3)	68(9)		
**t-ZrO**_**2**_	tetragonal *P*42/*nmc*	3.602(1)		5.181(2)		8.1(4)	42(1)		

In the case of SrZrO_3_ based-sample, the cubic SrZrO_3_, m-ZrO_2_ and t-ZrO_2_ phases were indexed. The quality parameters obtained were satisfactory for confirming the indexed phases. Similar to the previous system, the SrZrO_3_ phase is predominant, representing 84.4% of the system, while m-ZrO_2_ and t-ZrO_2_ correspond to 6.9 and 8.7%, respectively. The phases presented in this sample have similar crystallite sizes.

The XRD pattern of the SrZrO_3_/BaZrO_3_ composite (Figure 1c) is characterized by prominent peaks around 32, 43, 53, 62, and 71°, consistent with the diffraction data of cubic SrZrO_3_ and BaZrO_3_ phases with concentrations of 48.8 and 38.2%, respectively. All peaks exhibit well-defined crystal structures, indicating the formation of a composite with preserved crystallographic characteristics. This sample also presents the m-ZrO_2_ and t-ZrO_2_ phases. According to Table 1, the composite material presented a low percentage of m-ZrO_2_ and t-ZrO_2_ in comparison to SrZrO_3_ and BaZrO_3_ phases. Despite the low amounts of zirconium oxides in the sample, the zirconium oxides present in the composite may also contribute to the properties of the composite.

Figure 1d shows the infrared spectra obtained from 400 to 4000 cm^–1^ for the synthesized materials. The FTIR spectra obtained for SrZrO_3_ and BaZrO_3_ show a broad band in the range of 3100–3500 cm^–1^ and a band around 1600 cm^–1^, which can be attributed to the stretching and bending vibrations of the −OH groups of adsorbed water molecules, respectively.^18^ The small peaks around 2360 cm^–1^ observed in each mentioned sample can be attributed to atmospheric CO_2_.^19^ The peaks around 540 cm^–1^ are associated with the Zr–O stretching modes in zirconia, which aligns well with the XRD results since the samples exhibit a phase mixture.^18,19^ The FTIR spectrum of the SrZrO_3_/BaZrO_3_ composite followed the same trend as the individual materials, confirming the presence of phases of these materials.

The morphology of the BaZrO_3_ and SrZrO_3_/BaZrO_3_ samples was evaluated by scanning electron microscopy (SEM) as shown in Figure 2. The SEM image of the BaZrO_3_ (Figure 2a) showed cubic-shaped particles and other interconnected particles with irregular morphology. In the SEM image of the combined perovskites, SrZrO_3_/BaZrO_3_ (Figure 2b), it is possible to observe SrZrO_3_ particles distributed on BaZrO_3_ plates. The EDS spectrum (Figure 2c) shows the presence of Ba, Sr, O, and Zr elements in the SrZrO_3_/BaZrO_3_ sample, confirming the successful synthesis of the composite. The unexpected appearance of the sodium peak may have occurred due to the use of reagents in the synthesis of the perovskites that could contain sodium impurities, even in small amounts. The Au peak in the EDS spectrum is related to the Au film used for sample metallization. This metallization process is commonly used to enable scanning electron microscopy (SEM) analysis, as it reduces the accumulation of electrical charge on insulating samples and improves the quality of the images obtained.

**Figure 2:**
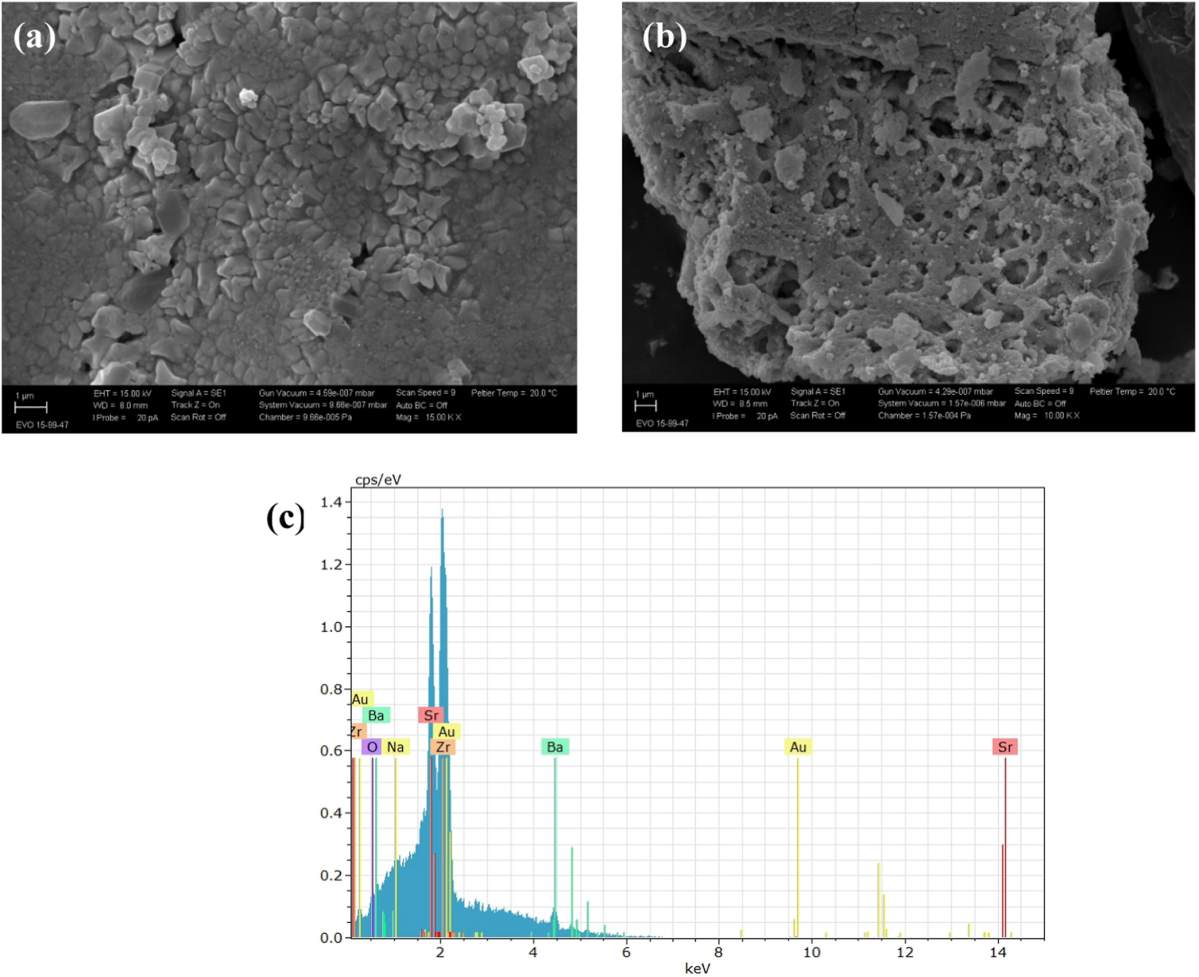
SEM images of the ceramic perovskite (a) BaZrO_3_ and (b) SrZrO_3_/BaZrO_3_. (c) EDS spectrum of SrZrO_3_/BaZrO_3_.

### 3.2 Photoelectrochemical Behavior of the SrZrO_3_/BaZrO_3_/FTO Platform

To investigate the photoelectrochemical activity of the materials used in the photoelectrochemical (PEC) platform, amperometric experiments were performed in the presence of 1000 μmol L^–1^ of DPE in 0.1 mol L^–1^ PBS (pH 7.0) and under an applied potential of 0.3 V vs Ag/AgCl/KCl_sat_. Photoelectrochemical signals were obtained for the electrodes modified with the individual components (SrZrO_3_/FTO and BaZrO_3_/FTO) as well as for the combined system (SrZrO_3_/BaZrO_3_/FTO platform) in absence and presence de DPE, as shown in Figure 3.

**Figure 3:**
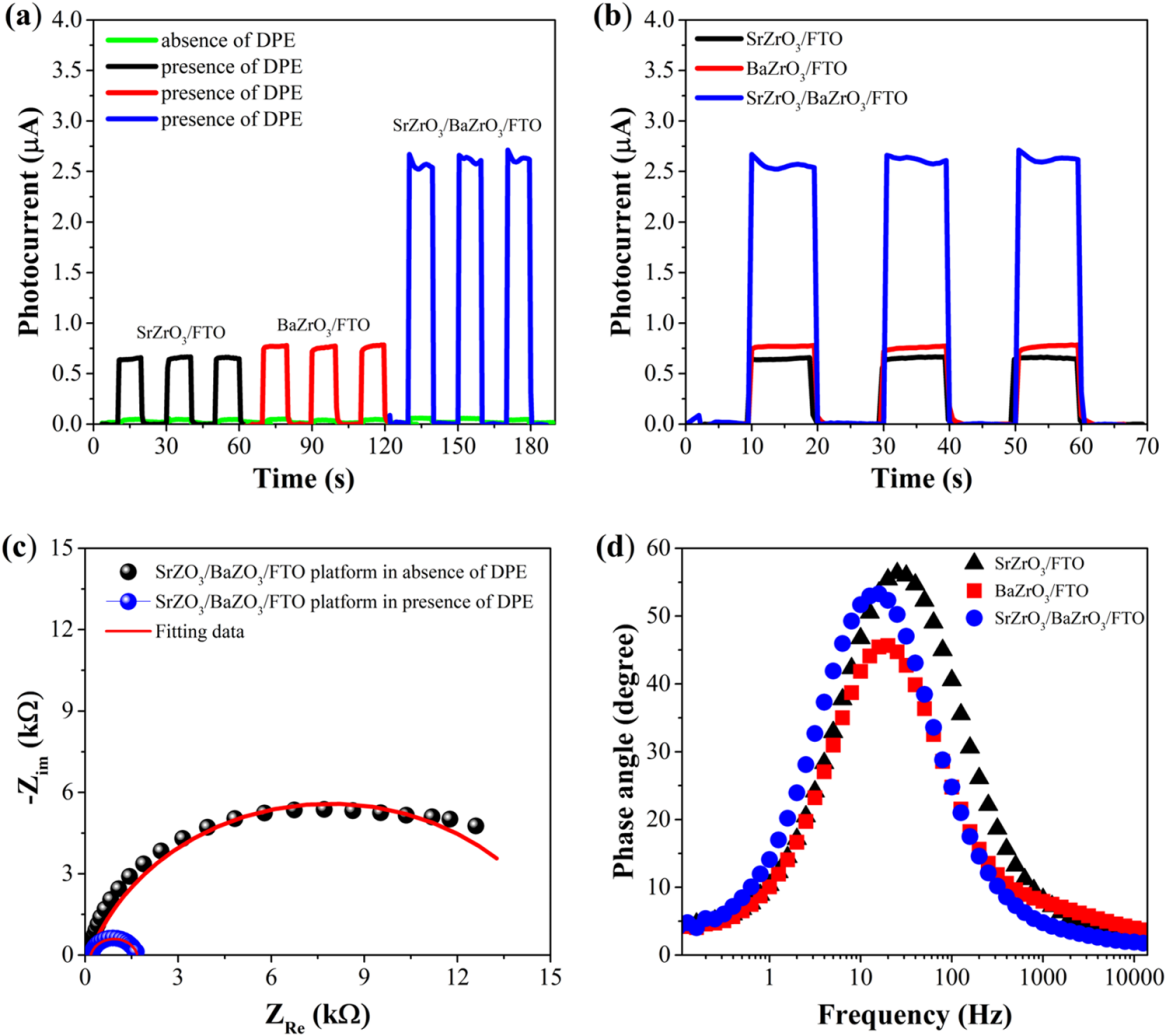
(a) Amperograms obtained for the SrZrO_3_/FTO, BaZrO_3_/FTO, and SrZrO_3_/BaZrO_3_/FTO platforms in the absence and presence of DPE. (b) Amperograms obtained for the SrZrO_3_/FTO, BaZrO_3_/FTO, and SrZrO_3_/BaZrO_3_/FTO platforms in the presence of DPE. The experiments were conducted in 0.1 mol L^–1^ PBS (pH 7.0) containing 1000 μmol L^–1^ DPE. (c) Nyquist plots of the SrZrO_3_/BaZrO_3_/FTO platform measured in 0.1 mol L^–1^ PBS in the presence of 1 mmol L^–1^ (blue spectrum) and absence DPE (black spectrum) under LED illumination, with a frequency range of 10 kHz to 0.1 Hz. (d) Bode-phase plots for SrZrO_3_/FTO, BaZrO_3_/FTO and SrZrO_3_/BaZrO_3_/FTO obtained in 0.1 mol L^–1^ PBS (pH 7.0) containing 1000 μmol L^–1^ DPE under incidence of light.

According to Figure 3a, in the absence of DPE, the three platforms (SrZrO_3_/FTO, BaZrO_3_/FTO, and SrZrO_3_/BaZrO_3_/FTO) exhibited very low photocurrents due to the recombination of photogenerated charges. However, in the presence of the analyte, the photocurrents increased significantly as the analyte donates electrons to the valence band of the semiconductor materials, thereby preventing the recombination. Results of Figure 3b revealed that the SrZrO_3_/BaZrO_3_/FTO platform demonstrated a significantly higher photocurrent compared to the SrZrO_3_/FTO and BaZrO_3_/FTO systems. This enhanced performance can be attributed to the synergistic interaction between SrZrO_3_ and BaZrO_3_, which improves the optical and electronic properties of the composite material. Both BaZrO_3_ and SrZrO_3_ are wide band gap materials, of approximately 5.0 and 5.6 eV, respectively.^12,13^ While these large band gaps typically limit their ability to absorb visible light, the interaction with DPE may introduce intermediate energy states within the band gap. This phenomenon is particularly important for enhancing the photocatalytic activity of the materials under visible light irradiation, as it allows for more efficient utilization of the light. The interaction between the dopamine and the perovskite surface can probably occur through the enediol ligands, which can exhibit affinity for uncoordinated surface sites allowing the Zr atoms to achieve a stable octahedral coordination, in a similar way observed for Ti-based semiconductors.^20^ This possible interaction can lead to the formation of a ligand-to-metal charge transfer complex, thereby enhancing the absorption of visible light and inhibiting the recombination of electron–hole pairs. In addition, the introduction of intermediate states within the energy gap, facilitated by the interaction with DPE, formation of disordered states as well as oxygen vacancies during the synthesis of raw materials may play a critical role in enhancing light absorption^21^ and charge separation. These findings highlight the promising character of this platform for the development of highly sensitive and selective photoelectrochemical sensors for the detection of biomolecules.

Figure 3c shows Nyquist plots of the SrZrO_3_/BaZrO_3_/FTO platform obtained in 0.1 mol L^–1^ PBS in the absence (black spectrum) and presence of 1000 μmol L^–1^ DPE (blue spectrum) under LED illumination, with a frequency range of 10 kHz to 0.1 Hz. These results show that in the presence of DPE, the PEC platform presented a smaller semicircle compared to the PEC platform in the absence of the analyte. The Nyquist spectra were fitted by performing the electrochemical circle fit using an equivalent circuit based on the solution resistance (Rs) in series with a parallel association of a resistor (Rct - charge transfer resistance) and a phase-constant element (PCE). The charge transfer resistances under light for the SrZrO_3_/BaZrO_3_/FTO platform in the absence and presence of DPE were 15.5 and 1.5 kΩ, respectively. These results indicate that the PEC platform is significantly affected by the presence of the DPE in the electrolyte solution. In order to evaluate the synergistic interaction between SrZrO_3_ and BaZrO_3_, it was investigated the lifetime of the excited electron (τ) of each platform. In this sense, Bode-phase plots for SrZrO_3_/FTO, BaZrO_3_/FTO and SrZrO_3_/BaZrO_3_/FTO were obtained in 0.1 mol L^–1^ PBS (pH 7.0) containing 1000 μmol L^–1^ DPE under incidence of light. The electron lifetime was obtained from the following equation:

2 $$\tau =1/2\pi f_{\max}$$

where *f*_max_ is the maximum frequency in the Bode phase plots. The values of maximum frequency for SrZrO_3_/FTO, BaZrO_3_/FTO and SrZrO_3_/BaZrO_3_/FTO photoelectrodes were 25.12, 19.95, and 15.85 Hz, such as the electron lifetimes to SrZrO_3_/FTO, BaZrO_3_/FTO and SrZrO_3_/BaZrO_3_/FTO were 6.34 ms, 7.98 and 10.04 ms, respectively. Thus, the corresponding electron lifetime in the SrZrO_3_/BaZrO_3_/FTO is larger than the other ones indicating a more effective separation of carriers produced in the SrZrO_3_/BaZrO_3_/FTO photoelectrochemical platform.

### 3.3 Optimization of Analytical Parameters of the SrZrO_3_/BaZrO_3_/FTO Platform

The optimization of analytical parameters for the SrZrO_3_/BaZrO_3_/FTO sensor represents a critical step in advancing the application of ceramic perovskite-based platforms for photoelectrochemical sensing. By systematically evaluating key experimental conditions, such as medium pH, buffer composition, and applied potential—this study identified optimal conditions that maximize the sensor’s photoelectrochemical performance.

The influence of solution pH on the sensor’s performance was investigated in the presence of 1000 μmol L^–1^ DPE, using 0.1 mol L^–1^ PBS as the buffer and an applied potential (*E*_appl_) of 0.3 V vs Ag/AgCl/KCl_sat_. The pH range tested (4.0 to 8.5) was selected to cover both mildly acidic and neutral conditions, which are relevant for many biological and environmental applications. Figure 4a presents the amperograms obtained at different pH values, while Figure 4b highlights the superior photoelectrochemical response observed at pH 7.0. This optimal response at neutral pH can be attributed to the stability of the perovskite material and the efficient charge transfer processes under these conditions. At lower pH values, protonation of surface sites may hinder electron transfer, while at higher pH values, the formation of hydroxyl species could compete with the redox reactions of DPE, reducing the sensor’s efficiency.

**Figure 4:**
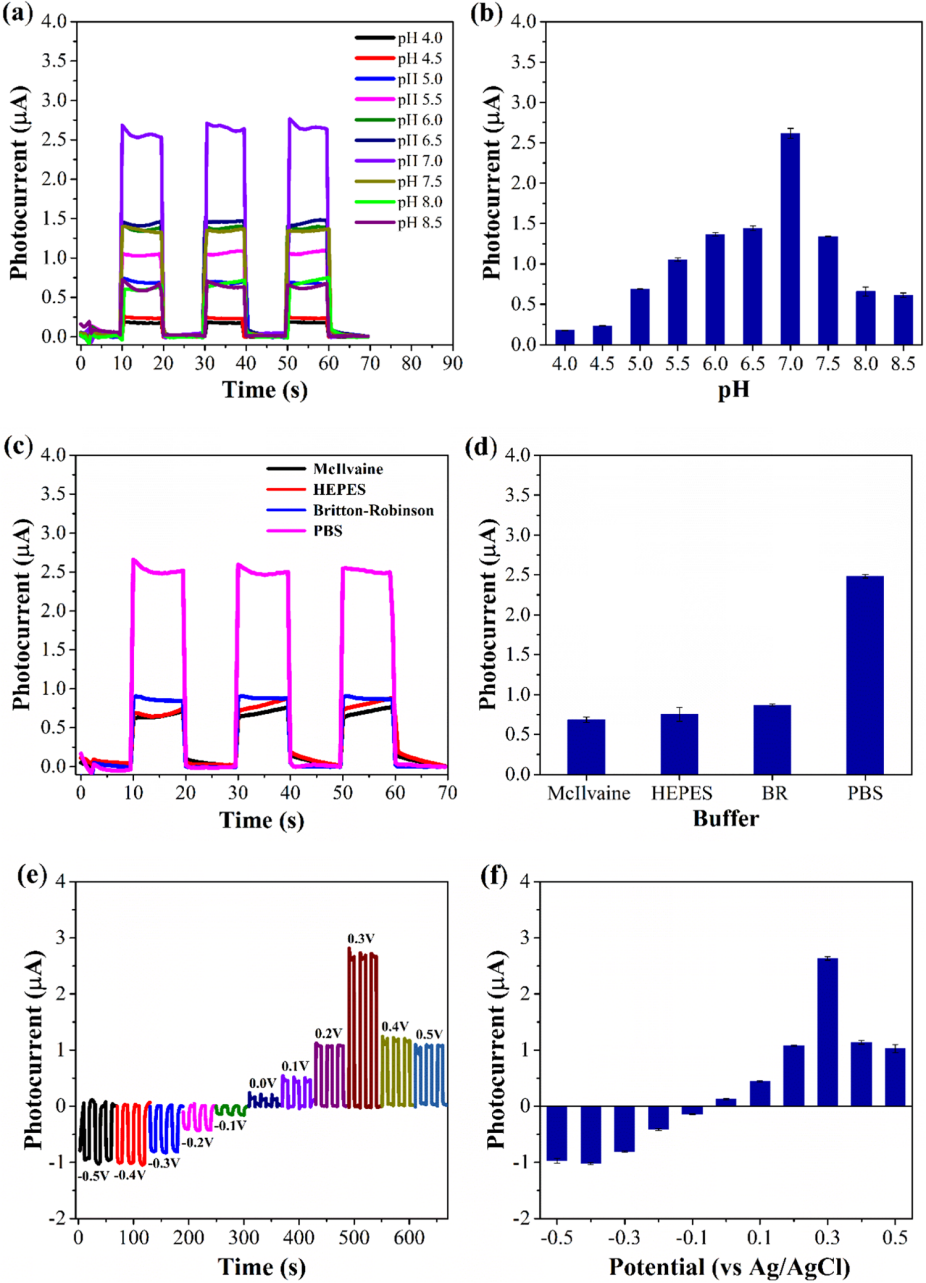
(a) Photoelectrochemical response of the SrZrO_3_/BaZrO_3_/FTO sensor as a function of pH in the range of 4.0 to 8.5 in the presence of 1000 μmol L^–1^ DPE in 0.1 mol L^–1^ PBS. (b) Graph of the average photocurrent value versus pH. (c) Photoelectrochemical response of the SrZrO_3_/BaZrO_3_/FTO sensor obtained in different buffers (0.1 mol L^–1^ and pH 7.0) in the presence of 1000 μmol L^–1^ DPE. (d) Graph of the average photocurrent value versus buffer solution type. (e) Photoelectrochemical response of the SrZrO_3_/BaZrO_3_/FTO sensor obtained at different potentials (*E*_appl._ = –0.5 to + 0.5 V vs Ag/AgCl/KCl_sat_) in the presence of 1000 μmol L^–1^ DPE in 0.1 mol L^–1^ PBS (pH 7.0). (f) Graph of the average photocurrent value versus potential.

The observed preference for neutral pH aligns with previous studies on perovskite-based sensors, which have demonstrated that neutral environments often enhance redox reactions by minimizing surface charge effects and stabilizing the photogenerated charge carriers. For instance, similar behavior has been reported for other perovskite materials, such as SrTiO_3_ and BaTiO_3_, where neutral pH conditions were found to optimize the balance between surface reactivity and material stability. This consistency across studies underscores the generalizability of the findings and highlights the importance of pH control in photoelectrochemical sensing.

The photoelectrochemical response of the SrZrO_3_/BaZrO_3_/FTO sensor was evaluated in four types of buffers (PBS, Britton-Robinson, McIlvaine, and HEPES) (Figure 4c). Figure 4d shows the photocurrent values for these buffer solutions. It is observed that the buffer solution providing the best photocurrent response to DPE was PBS, which was therefore used in the subsequent experiments. Moreover, the choice of PBS as the buffer solution further supports the sensor’s performance, as PBS provides a stable ionic environment that facilitates efficient charge transfer. The effect of the applied potential on the response of the SrZrO_3_/BaZrO_3_/FTO sensor in the presence of 1000 μmol L^–1^ DPE was also investigated, and the results are presented in Figure 4e. According to the results, it is observed that the photocurrent increases from 0 to 0.3 V vs Ag/AgCl/KCl_sat_ and decreases beyond this potential. Furthermore, at potential values lower than 0 V vs Ag/AgCl/KCl_sat_, the currents are negative and increase from −0.1 to −0.4 V vs Ag/AgCl/KCl_sat_, remaining constant at −0.5 V vs Ag/AgCl/KCl_sat_, but remain lower than the photocurrent obtained at 0.3 V vs Ag/AgCl/KCl_sat_. At this potential (0.3 V), charge separation and electron transfer are at their most efficient state, maximizing the photocurrent, therefore, the applied potential of 0.3 V vs Ag/AgCl/KCl_sat_ was fixed in subsequent studies. These results collectively demonstrate the importance of fine-tuning experimental parameters to achieve the best possible performance from perovskite-based sensors.

In conclusion, the optimization of pH, buffer composition, and applied potential has significantly enhanced the photoelectrochemical response of the SrZrO_3_/BaZrO_3_/FTO sensor. The findings not only show the potential of perovskite-based materials for sensing applications but also provide valuable insights into the interplay between material properties and experimental conditions. Future work could explore the sensor’s performance in real-world samples, such as environmental or biological fluids, to further validate its practical applicability.

### 3.4 Analytical Performance of the SrZrO_3_/BaZrO_3_/FTO Platform and Its Application in Artificial Plasma Samples

The precision of the SrZrO_3_/BaZrO_3_/FTO sensor was evaluated through successive photocurrent measurements conducted in a single day and over five different days. Experiments were carried out in 0.1 mol L^–1^ PBS (pH 7.0) under an applied potential of 0.3 V vs Ag/AgCl/KCl_sat._ For the intraday test, amperometric measurements were performed using a concentration of 1000 μmol L^–1^ DPE over 500 s, with 25 photocurrent recordings (Figure 5a). The photocurrents remained stable, resulting in a relative standard deviation (RSD) of 2.6%, indicating good repeatability of the response of the PEC sensor. Additionally, five sensors were evaluated over five different days in the presence of 1000 μmol L^–1^ DPE to verify method reproducibility (Figure 5b), yielding an RSD of 3.8%, which demonstrates good reproducibility of the response of the PEC sensor. The low standard deviations observed reinforce the sensor’s precision.

**Figure 5:**
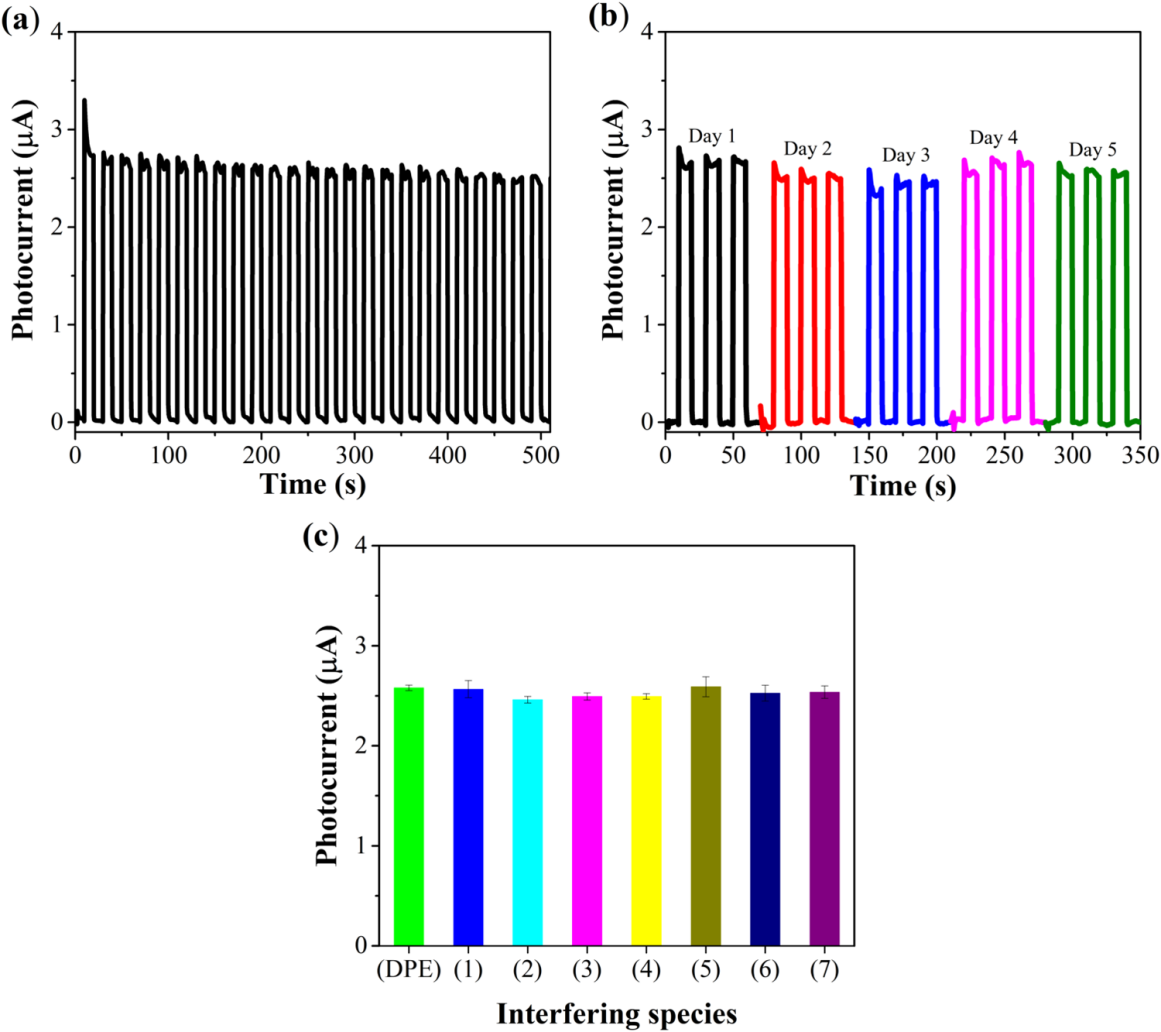
(a) Photocurrents measured with the SrZrO_3_/BaZrO_3_/FTO sensor in the presence of 1000 μmol L^–1^ DPE over 500 s. (b) Photocurrents obtained by the SrZrO_3_/BaZrO_3_/FTO sensor in the presence of 1000 μmol L^–1^ DPE on different days. (c) Evaluation of the influence of interfering species on DPE detection: (1) sucrose, (2) ascorbic acid, (3) magnesium chloride, (4) sodium sulfite, (5) citric acid, (6) glucose, (7) fructose, with DPE and interferent concentrations fixed at 1000 μmol L^–1^. The experiments were conducted in 0.1 mol L^–1^ PBS (pH 7.0), with an applied potential of 0.3 V vs Ag/AgCl/KCl_sat_.

To assess the interference from potentially interfering species, the response of the SrZrO_3_/BaZrO_3_/FTO sensor was measured in the presence of 1000 μmol L^–1^ DPE and 1000 μmol L^–1^ of each interfering substance (sucrose, magnesium chloride, ascorbic acid, sodium sulfite, citric acid, glucose, and fructose). The selectivity study results (Figure 5c) showed that the analytical signal did not vary significantly, indicating that these species do not interfere with DPE detection, highlighting the good selectivity of the SrZrO_3_/BaZrO_3_/FTO sensor.

Figure 6a shows the amperograms for different DPE concentrations (0.01 to 500 μmol L^–1^). An increase in the system’s photocurrent is observed as the DPE concentration increases, showing a linear response in two concentration ranges: 0.01 to 100 μmol L^–1^ and 100 to 500 μmol L^–1^ (Figure 6b). The linear regression equations for the analytical curves presented in Figure 6b are

3 $$I/\mu A=0.12+0.012\;\left[\mathrm{DPE}\right]\;\mu {\mathrm{mol}\;L}^{-1}\;\left(r=0.994\right)$$

4 $$I/\mu A=1.05+0.002\;\left[\mathrm{DPE}\right]\;\mu {\mathrm{mol}\;L}^{-1}\;\left(r=0.998\right)$$

**Figure 6:**
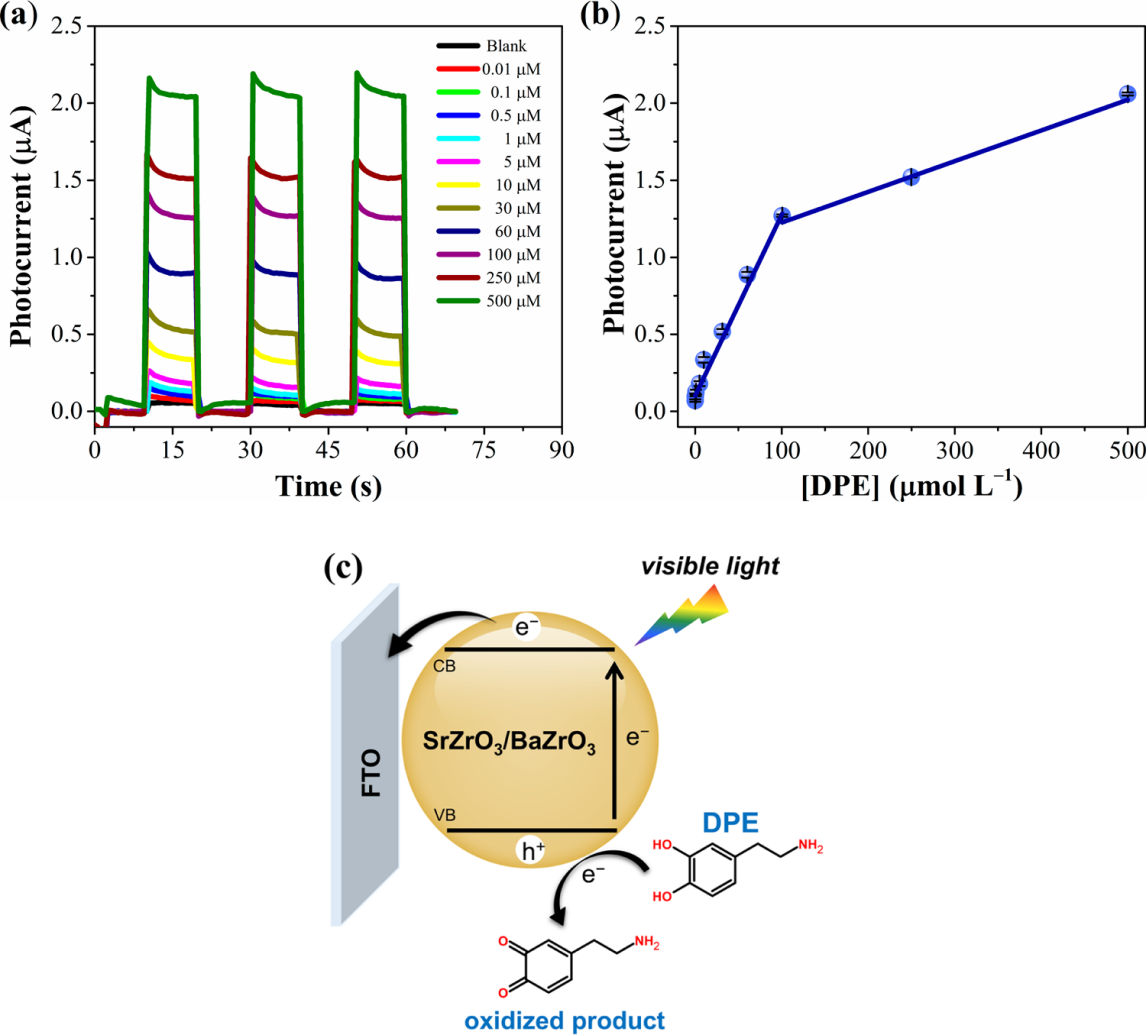
(a) Photoelectrochemical response of the SrZrO_3_/BaZrO_3_/FTO sensor at different DPE concentrations (0.01, 0.1, 0.5, 1.0, 5.0, 10, 30, 60, 100, 250, and 500 μmol L^–1^) obtained in 0.1 mol L^–1^ PBS (pH 7.0), *E*_appl._ = 0.3 V. (b) Plot of the photocurrent as a function of DPE concentration (*n* = 3). (c) Representative scheme of the photoelectrochemical detection mechanism of DPE using the SrZrO_3_/BaZrO_3_/FTO sensor.

The limit of detection (LOD) found at 0.009 μmol L^–1^ (*n* = 3) was calculated using the formula LOD = 3 × SD/b (5), where SD is the standard deviation and b is the slope of the analytical curve. The detection limit and linear range data obtained in this study were compared with other analytical methods developed for DPE determination, as shown in Table 2. Compared to other detection methods, the proposed method offers significant advantages in terms of linear range and detection limit.

**Table 2:** Comparison of Analytical Parameters of Different Methods for DPE Determination

method	linear range (μmol L^–1^)	LOD (μmol L^–1^)	refs
LSV (δ-MnO_2_/S@gC_3_N_4_/SPCE)^a^	3.0–125	1.12	^22^
PEC BiVO_4_/BiOCl^b^	1–13	0.134	^23^
DPV (CuO NFs/GCE)^c^	0.1–800	0.03	^24^
DPV (Ni-MOF/GCE)^d^	0.2–100	0.06	^25^
fluorescence	1.0–200	0.3	^26^
colorimetry	0.1–10	0.04	^27^
DPV (N-GSs/GCE)^e^	0.1–700	0.03	^28^
PEC (SrZrO_3_/BaZrO_3_/FTO)	0.01–100 and 100–500	0.009	this work

a Manganese dioxide composed with sulfur-doped graphitic carbon nitride using linear sweep voltammetry.

b Chlorine oxide electrode based on bismuth composed with bismuth vanadate through photoelectrochemical method.

c Glassy carbon electrode modified with copper oxide nanoflowers using differential pulse voltammetry.

d Glassy carbon electrode modified with nickel-based metal–organic framework using differential pulse voltammetry.

e Glassy carbon electrode modified with nitrogen-doped graphene sheets using differential pulse voltammetry.

Figure 6c presents a proposed scheme for DPE detection using the PEC sensor based on SrZrO_3_/BaZrO_3_/FTO under visible LED illumination. Upon absorbing visible light, BaZrO_3_ transfers electrons from its valence band (VB) to the conduction band (CB). Simultaneously, pairs of electrons (e^–^) and holes (h^+^) are generated. These electrons migrate to the CB of SrZrO_3_ and are collected by the FTO electrode. DPE, under visible light excitation, is involved in electron transfer at the electrode surface, capturing photogenerated holes (h^+^) in the VB of BaZrO_3_, inhibiting charge recombination of electron–hole pairs, and increasing the photocurrent response.^29^

The developed SrZrO_3_/BaZrO_3_/FTO sensor was applied to artificial plasma samples enriched with DPE at concentrations of 5, 10, and 100 μmol L^–1^. Quantification was performed using external calibration. As shown in Table 3, the recovery values ranged from 98.32 to 102.13%, with low relative standard deviations, demonstrating the high accuracy and precision of the sensor. This clearly shows that the sensor can efficiently detect and quantify DPE in plasma.

**Table 3:** Determination of DPE in Artificial Plasma Samples

samples	[DPE] added (μM)	[DPE] found (μM)	recovery (%)	RSD (%)
1	5	4.916	98.32	3.1
2	10	10.143	101.43	1.3
3	100	102.13	102.13	1.9

## 4 Conclusions

The developed SrZrO_3_/BaZrO_3_/FTO sensor exhibited good visible light response demonstrating that the application of these two perovskites to determination of DPE can result in an excellent performance. The LED light used in this study is a safe and sustainable choice, as it does not emit harmful radiation and has low power consumption. The SrZrO_3_/BaZrO_3_ combination demonstrated high performance by minimizing the recombination of photoinduced charge carriers, thus enhancing the efficiency of the SrZrO_3_/BaZrO_3_ composite material compared to its individual components.

The SrZrO_3_/BaZrO_3_/FTO sensor showed remarkable performance for DPE detection, standing out for its high selectivity and broad linear response range from 0.01 to 100 μmol L^–1^ and 100 to 500 μmol L^–1^. This sensor demonstrated excellent accuracy and precision, along with a low detection limit of 0.009 μmol L^–1^, allowing for the detection of DPE at low concentrations. These characteristics suggest the potential of this sensor as an effective and sensitive tool for monitoring DPE in biological samples, contributing to advances in the diagnosis of various diseases. Thus, the combination of BaZrO_3_ with SrZrO_3_ enabled the development of materials with optimized properties for photoelectrochemical applications, resulting in high photoelectrochemical performance, chemical stability, and ease of synthesis, making it an excellent alternative for the detection and quantification of DPE.
